# A systematic review and meta-analysis of Danshen combined with mesalazine for the treatment of ulcerative colitis

**DOI:** 10.3389/fphar.2024.1334474

**Published:** 2024-05-31

**Authors:** Wei Zhang, Peiyu Xiong, Junyu Liu, Hengchang Hu, Li Song, Xinglong Liu, Bo Jia

**Affiliations:** College of Basic Medicine, Chengdu University of Traditional Chinese Medicine, Chengdu, Sichuan, China

**Keywords:** Danshen, Danshen preparation, mesalazine, ulcerative colitis, meta-analysis, systematic review

## Abstract

**Purpose:** Current pharmacological treatments for Ulcerative Colitis (UC) have limitations. Therefore, it is important to elucidate any available alternative or complementary treatment, and Chinese herbal medicine shows the potential for such treatment. As a traditional Chinese herbal medicine, Danshen-related preparations have been reported to be beneficial for UC by improving coagulation function and inhibiting inflammatory responses. In spite of this, the credibility and safety of this practice are incomplete. Therefore, in order to investigate whether Danshen preparation (DSP) is effective and safe in the treatment of UC, we conducted a systematic review and meta-analysis.

**Methods:** PubMed, Embase, Cochrane Library, Web of Science, China National Knowledge Infrastructure, Wanfang Database and CQVIP Database were searched for this review.The main observation indexes were the effect of DSP combined with mesalazine or DSP on the effective rate, platelet count (PLT), mean platelet volume (MPV) and C-reactive protein (CRP) of UC. The Cochrane risk of bias tool was used to assess the risk of bias. The selected studies were evaluated for quality and data processing using RevMan5.4 and Stata17.0 software.

**Results:** A total of 37 studies were included. Among them, 26 clinical trials with 2426 patients were included and 11 animal experimental studies involving 208 animals were included. Meta-analysis results showed that compared with mesalazine alone, combined use of DSP can clearly improve the clinical effective rate (RR 0.86%, 95% CI:0.83–0.88, *p* < 0.00001) of UC. Furthermore it improved blood coagulation function by decreasing serum PLT and increasing MPV levels, and controlled inflammatory responses by reducing serum CRP, TNF-α, IL-6, and IL-8 levels in patients.

**Conclusion:** Combining DSP with mesalazine for UC can enhance clinical efficacy. However, caution should be exercised in interpreting the results of this review due to its flaws, such as allocation concealment and uncertainty resulting from the blinding of the study.

**Systematic Review Registration**: http://www.crd.york.ac.uk/PROSPERO/myprospero.php, identifier PROSPERO: CRD42022293287

## 1 Introduction

Ulcerative colitis (UC) is an inflammatory bowel disease primarily affecting the rectum and colon mucosa, characterized by abdominal pain, diarrhea, and mucopurulent stools as its main symptoms. UC poses serious health and safety risks to patients, significantly impacting their quality of life and increasing mortality (Ungaro et al., 2017). Classified by the World Health Organization as one of the most challenging diseases to treat, UC is difficult to cure and prone to recurrence and cancer. While primarily found in Western countries, its incidence is on the rise in developing nations (Ramos and Papadakis, 2019). There is no sex predilection in UC, with the peak age of onset occurring between 30 and 40 years old (Ungaro et al., 2017). However, its exact etiology remains not fully understood. Conventional anti-inflammatory agents like sulfasalazine and mesalazine have a long history of use, alongside bioimmunosuppressive agents, steroids, and microbiome therapies, which have also shown efficacy (Plichta et al., 2019). Although numerous drugs are available for UC treatment, not all patients respond to these medications, and some carry significant adverse effects. Therefore, efforts to enhance UC treatment efficacy are imperative.The traditional Chinese medicinal herb Danshen, with a history of over 2,000 years, consists of the dried root and rhizome of *Salvia miltiorrhiza Bge.* Danshen is primarily produced in Hebei, Shanxi, Shandong, and other regions of China. The 2020 edition of the Chinese Pharmacopoeia records that Danshen can activate blood circulation, remove blood stasis, clear heat from the blood, and tranquilize the mind (National Pharmacopoeia Commission, 2020). Consequently, it has traditionally been used to treat various conditions such as cerebrovascular hemorrhage, edema, malignancies, menstrual abnormalities, miscarriages, and cardiovascular diseases (Nwafor et al., 2021). Modern studies have revealed that Danshen exhibits a wide range of complex pharmacological effects. Its chemical components mainly include diterpenoids, triterpenoids, phenolic acids, flavonoids, nitrogenous compounds, lactones, and polysaccharides (Mei et al., 2019; Li et al., 2020; Wan et al., 2020). Among these, diterpenoids and phenolic acids are the primary active components of Danshen. Danshen demonstrates pharmacological effects such as anti-tumor, anti-inflammatory, immunomodulatory, anti-fibrosis, and cardiovascular benefits (Shan et al., 2021).

Due to the diverse structures and extensive pharmacological effects of the active components of Danshen, many scholars have initiated studies on Danshen preparations (Li et al., 2022; Shen et al., 2022). These preparations include Danshen acid B dry powder (Lu et al., 2022), Danshen injection (Wang et al., 2017), Tanshinone capsules (Fu et al., 2022), Sodium Tanshinone IIA Sulfonate (Zhou et al., 2021), and others, which have been widely used in treating various diseases. The anti-inflammatory effects of Danshen extracts and related preparations have been extensively investigated (Yuan et al., 2021). Studies have revealed that Danshen may play a therapeutic role in UC by inhibiting platelet activation (Zhang, 2017), improving blood hypercoagulability (Liu et al., 2011a), antagonizing oxidative damage (Fu et al., 2023), enhancing intestinal flora (Wang et al., 2018a), and regulating inflammatory factors (Min et al., 2020; Peng et al., 2021a). In recent years, Danshen preparations have been widely utilized as adjuvants in UC treatment, with numerous animal and clinical trials demonstrating the reliable efficacy of Danshen in treating ulcerative colitis (Liu et al., 2019; Liu et al., 2020; Wu et al., 2021). However, there remains a lack of reliable evidence-based data to evaluate the efficacy and safety of Danshen. In this study, we comprehensively collected published literature and utilized meta-analysis to systematically evaluate the efficacy and safety of Danshen combined with mesalazine or used alone in UC treatment, employing a large, objective sample size, aiming to provide higher-level evidence for clinical decision-making.

## 2 Methods and materials

Based on the Cochrane Handbook for Systematic Reviews of Interventions, the meta-analysis and systematic review were performed according to PRISMA (Preferred Reporting Items for Systematic Reviews and Meta-analyses) (Page et al., 2021) and PRISMA-CHM 2020 (PRISMA Extension for Chinese Herbal Medicines 2020) guidelines.

### 2.1 Protocol and registration

The systematic review and meta-analysis has been registered (Registered with PROSPERO: CRD42022293287, available online at http://www.crd.york.ac.uk/PROSPERO/myprospero.php.

### 2.2 Search strategy

We used “Dan Shen” and “Ulcerative Colitis” as search terms to search various literature and electronic databases for randomized controlled trials from January 2000 to May 2023. These sources included PubMed, Embase, Web of Science, CNKI, Wanfang Database and CQVIP Database.

In particular, medical subject headings (MeSH) terms with free words were employed in English databases. The relevant terms were as follows: ulcerative colitis [MeSH], UC, ulcer colonitis, colitis gravis; DanShen [MeSH], Tan Seng, Chinese Salvia. Chinese databases were searched according to the aforementioned search terms in Chinese. The detailed search strategies containing more search terms are provided in Supplementary Material S1.

We also searched the International Clinical Trial Registry (http://ClinicalTrials.gov/and the Chinese Clinical Trial Registry (Http://www.chictr.org.cn/index.aspx.

### 2.3 Inclusion and exclusion criteria

We review the inclusion criteria: 1) Participants: had a diagnosis of UC, regardless of gender or ethnicity and all animal models with UC; 2) Intervention: DSP combined with mesalazine was used in the clinical study, and DSP was used in the animal experiment, the amount of herbal medicine in DSP conforms to the standard stipulated by Chinese Pharmacopoeia (2020 edition); 3) Control group: mesalazine alone, same solvent, no intervention, etc.; 4) Study design: randomized controlled trials; 5) Outcomes: effective rate, PLT, MPV and CRP were the primary outcomes, TNF-α, IL-6 and IL-8 were the secondary outcomes; 6) Language: Chinese and English.

We review the exclusion criteria: 1) Duplicate publication; 2) Study design: traditional systematic reviews, case reports, guidelines, recommendations, etc.; 3) *in vitro* studies, etc.; 4)Not an original full research paper (e.g., conference proceedings, review, abstracts); 5) Control group: other medications.

### 2.4 Study selection and data extraction

**FIGURE 1:**
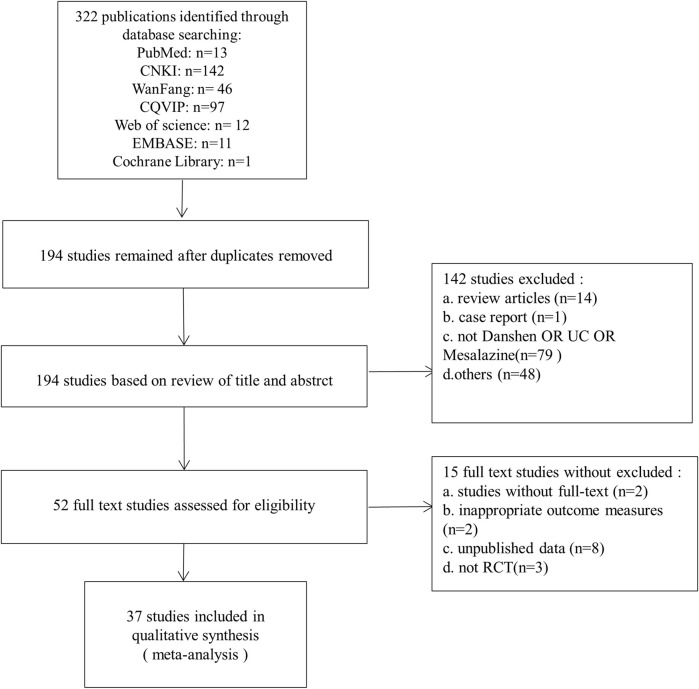
Flow diagram of the study selection process for this review.

The selection of material was first screened by 2 reviewers who read the title and abstract of the article independently. The two reviewers then read the full text separately to determine whether the final article should be included. At each stage, the two reviewers would independently record the results of the screening. In the event of disagreement, the two reviewers would negotiate a settlement or a third reviewer would be consulted. The integration process is shown as PRISMA flow chart (Figure 1).

A data collection table was designed in advance, with two reviewers independently extracting and collecting data from the articles involved, including the following contents: 1) Basic information: title, author and year; 2) Baseline data: sample size, age, sex, species, etc.; 3) Intervention information: Figure 15 Traditional Chinese medicine preparation, control intervention medication, intervention time; 4) Outcome measures: effective rate, PLT, MPV, CRP, TNF-α, IL-6 and IL-8. For continuous data (TNF-α, IL-6, IL-8), the mean and the standard deviation of each intervention group were extracted. For dichotomous data (e.g., effective rate), the numbers experiencing the outcome and the total numbers for each intervention group were collected. If a trial contains more than two intervention groups, interventions that meet the eligibility criteria will be included in the review (Higgins and Green, 2011).

### 2.5 Quality assessment

Two reviewers assessed the quality of the included clinical studies using Cochrane risk of bias tool (RoB-2 tool). The RoB-2 tool contains 5 entries based on six types of bias: 1) the randomization process; 2) deviations from intended interventions; 3) missing outcome data; 4) measurement of the outcome; and 5) selection of the reported result. The judgments are expressed simply as “low,” “high” or “some concerns” of bias (Sterne et al., 2019).

The methodological quality of included animal studies was evaluated on the basis of the SYRCLE’s RoB tool. The SYRCLE’s RoB tool for animal experiments involves 10 entries based on six types of bias: 1) Sequence generation (selection bias); 2) Baseline characteristics (selection bias); 3) Allocation concealment (selection bias); 4) Random housing (performance bias); 5) Blinding (performance bias); 6) Random outcome assessment (detection bias); 7) Blinding (detection bias); 8) Incomplete outcome data (attrition bias); 9) Selective outcome reporting (reporting bias); (10) Other sources of bias (other). The results of the evaluation are “yes,”“no” and “unclear,” representing “low risk of bias,”“high risk of bias” and “insufficient details have been reported to assess the risk of bias properly” (Hooijmans et al., 2014).

Two reviewers independently assessed the risk of bias. If there is a difference in assessment results, two reviewers should consult or consult with a third party.

### 2.6 Statistical analysis

This systematic review used RevMan 5.4 (developed by the Cochrane Collaboration international, United Kingdom) software and Random-effects (DerSimonian and Laird) method to analyze the data. The details are as follows: 1) Outcome measures: The standardized mean difference (SMD) was considered for continuous data (e.g., TNF-α, IL-6), while the pooled effect size was expressed as risk ratio (RR) for dichotomous data. 2) Statistically significant: The confidence interval (CI) was established at 95%, and *p*-value <0.05 was considered to be statistically significant. 3) Between-study heterogeneity: The chi-squared test with a significance level of α = 0.1 was used as statistical measure of heterogeneity, I^2^ > 50% represented a substantial heterogeneity. 4) Subgroup analyses: According to the intervention duration (<1 month, 1 month, >1 month), composition (DanShen, tanshinone), species (rat, mice) and DanShen application dose, to explore the potential sources of heterogeneity and the influence of various factors on the combined effect size. 5) Sensitivity analysis: Following each document’s exclusion, the new results were re-analyzed and compared with the original results to determine if the results remained stable.

### 2.7 Assessment of reporting bias

In the case of meaningful outcome indicators where at least 10 studies were included, a funnel plot was used to detect publication bias qualitatively. Begg’s test (Begg and Mazumdar, 1994) and Egger’s test (Egger et al., 1997) were used to evaluate potential publication bias quantitatively.

## 3 Results

### 3.1 Literature search

After retrieval, a total of 322 studies were included, 128 of which were deleted due to duplication. After reading the titles and abstracts of the 194 studies, 142 of them did not meet the requirements and were therefore excluded. The reasons for exclusion were as follows: 1) 1 study was case report; 2) 14 studies were experimental study; 3) 79 studies were not Danshen or UC or mesalazine; 4) 48 other studies. The full text of 52 studies was read with reference to inclusion and exclusion criteria for inclusion in the systematic review, 15 studies were excluded for lack of sufficient data. Eventually, 37 studies (Chen et al., 2005; Liu (2011); Xin et al., 2015; Xu, 2015; Zhang et al., 2015; Deng et al., 2016; Li, 2016; Xiong and Wang, 2016; Liang et al., 2017; Yang and Wang, 2017; Zhou et al., 2017; He, 2018a; He, 2018b; Wang et al., 2018b; Chen et al., 2018; Guo et al., 2018; Liu et al., 2018; Zhu, 2018; Du, 2019; Li, 2019; Li and Tian, 2019; Sun, 2019; Zhu, 2019; Wang, 2020a; Liu et al., 2020; Wang, 2020b; Xu, 2020; Zhang, 2020; Peng et al., 2021b; Wu et al., 2021; Su et al., 2021; Zhao, 2021; Zhu et al., 2021; Fan et al., 2022; Wang, 2022; Zhou, 2022; Zhu et al., 2022) were included in China formed eligible for systematic review, and PRISM flow chart is shown in Figure 1.

### 3.2 Study characteristics

The main characteristics of the included clinical studies are shown in Table 1, and the animal experimental studies are shown in Table 2. Studies reported specific outcomes in Table 3. All the 37 studies were randomized controlled trials. Among them, 26 were clinical trials and 11 were animal experiments. The clinical studies involved a total of 2426 participants, 1213in the intervention group and 1213 in the control group. Participants in all studies were both male and female and were from China. Participants were 18–70 years of age in 25 studies, and age was not reported in one study. Participants were recruited from the inpatient or outpatient departments of their respective institutions and all met internationally accepted diagnostic criteria for UC (Winther et al., 1998). In all studies, DSP included two chemical components, namely, DanShen and tanshinone.The treatment strategy for the intervention group was DSP combined with mesalazine and for the control group was mesalazine alone. The duration of treatment ranged from 7 days to 2 months.

**TABLE 1:** Characteristics of the included clinical studies.

Author	Sample size (E/C)	Male to female ratio (E/C)	Age (years) (m ± sd) (E/C)	Disease duration (years) (m ± sd) (E/C)	Experimental (E)	Control (C)	Treatment duration	Outcomes measures
Chen. (2018)	78 (39/39)	23:26/19:20	47.13 ± 6.61/46.48 ± 6.34	5.67 ± 3.24/5.41 ± 3.67	Mesalazine + DanShen injection (20 mL,qd, ivgtt)	Mesalazine (0.4g, tid,po)	2weeks	1/2
Deng. (2016)	110 (55/55)	25:30/23:32	58.3 ±8.2/57.6 ± 7.5	4.8 ± 1.4/4.5 ± 1.3	Mesalazine + DanShen injection (20 mL,qd, ivgtt)	Mesalazine (1g, tid,po)	4weeks	1/2/3/5/6
Du. (2019)	72 (36/36)	20:16/19:17	44. 2 ± 2. 7/44. 6 ± 2. 5	3.3 ± 1.4/3.2 ± 1.2	Mesalazine + DanShen injection (4 mL,qd/bid, ivgtt)	Mesalazine (0.5-1g, qid,po)	1month	1/3/5/6
He. (2018a)	110 (55/55)	29:26/31:24	44.56 ± 10.11/45.88 ± 9.22	8.06 ± 4.83/7.21 ± 5.30	Mesalazine + Tanshinone capsules (1g, tid,po)	Mesalazine (0.8g, tid,po)	28days	4/5/6
He. (2018b)	88 (44/44)	24:20/26:18	44.56 ± 10.11/45.88 ± 9.22	8.06 ± 4.83/7.21 ± 5.30	Mesalazine + Tanshinone capsules (1g, tid,po)	Mesalazine (0.8g, tid,po)	28days	4/5/6
Li. (2016)	120 (60/60)	68:52	41.38 ± 8.26/41.38 ± 8.26	3.72 ± 3.34/3.72 ± 3.34	Mesalazine + DanShen injection (20 mL,qd, ivgtt)	Mesalazine (1g, tid,po)	4weeks	1/3/5/6
Li. (2019)	86 (43/43)	23:20/21:22	44.18 ± 3.54/43.58 ± 5.12	6.41 ± 1.32/6.59 ± 1.47	Mesalazine + Tanshinone ⅡA injection (80 mg,qd, ivgtt)	Mesalazine (1000mg, qid,po)	7days	1/2
Liang. (2017)	120 (60/60)	34:26/25:35	53.2 ± 10.8/55.1 ± 11.7	6.1 ± 1.5/6.3 ± 1.2	Mesalazine + DanShen injection (20 mL,qd, ivgtt)	Mesalazine (1g, qid,po)	30days	1/2/3/5/6
Li and Tian, 2019	102 (51/51)	60:42	49.3 ± 6.7/49.3 ± 6.7	Unclear	Mesalazine + DanShen powder injection (0.4 g,qd, ivgtt)	Mesalazine (1g, tid,po)	8weeks	1/2/3
Liu (2011)	233 (116/117)	53:47/58:42	49.53 ± 8.16/47.95 ± 7.64	Unclear	Mesalazine + DanShen powder injection (0.4 g,qd, ivgtt)	Mesalazine (1g, tid,po)	Unclear	5
Liu. (2018)	82 (41/41)	26:15/25:16	47.54 ± 5.15/48.11 ± 5.44	4.68 ± 1.65/4.51 ± 1.51	Mesalazine + Tanshinone capsules (1g, tid,po)	Mesalazine (0.8g, tid,po)	2months	5/6
Sun. (2019)	86 (43/43)	23:20/25:18	45.6 ± 1.2/45.4 ± 1.3	2.3 ± 0.4/2.4 ± 0.3	Mesalazine + DanShen injection (4 mL,qd/bid, ivgtt)	Mesalazine (1g, tid/qid,po)	1month	4
Wang. (2018a)	120 (60/60)	28:32/29:31	50.2 ± 9.8/51.0 ± 10.7	5.8 ± 1.2/5.6 ± 1.1	Mesalazine + DanShen powder injection (0.4 g,qd, iv)	Mesalazine (1g, tid,po)	18days	1/2/3/5
Wang, 2020a	92 (46/46)	33:13/32:14	38.34 ± 5.93/37.60 ± 5.80	4.21 ± 0.96/4.23 ± 0.87	Mesalazine + DanShen injection (20 mL,qd, ivgtt)	Mesalazine (1g, tid,po)	1month	1/3/5
Wang. (2020b)	78 (39/39)	20:19/21:18	45.1 ± 6.31/44.87 ± 5.62	3.09 ± 1.24/3.07 ± 1.18	Mesalazine + DanShen injection (20 mL,qd, ivgtt)	Mesalazine (0.5g, tid,po)	1month	5/6
Wang. (2022)	95 (48/47)	25:23/25:22	50.63 ± 6.79/50.42 ± 6.71	3.68 ± 0.83/3.07 ± 1.18	Mesalazine + DanShen injection (20 mL,qd, ivgtt)	Mesalazine (1g, tid,po)	1month	1/2/3/5/6
Xin. (2015)	100 (50/50)	17:18/19:16	42.46 ± 2.19/41.73 ± 3.01	6.97 ± 0.94/7.01 ± 1.32	Mesalazine + Tanshinone IIA Sulfonate Injection (80mg, qd, ivgtt)	Mesalazine (1g, qid,po)	7days	4
Xiong and Wang. (2016)	70 (35/35)	16:19/15:20	47.4 ± 6.2/46.8 ± 7.1	5.1 ± 2.3/5.2 ± 2.1	Mesalazine + Tanshinone capsules (1g, tid,po)	Mesalazine (1g, tid,po)	8weeks	1/2/4/5/6
Xu. (2015)	84 (42/42)	25:17/23:19	44.1 ± 10.1/44.8 ± 9.7	6.0 ± 1.8/6.3 ± 1.7	Mesalazine + Tanshinone capsules (1g, tid,po)	Mesalazine (0.8g, tid,po)	8weeks	4/5
Xu. (2020)	88 (44/44)	25:19/24:20	39.50 ± 3.50/39.00 ± 3.50	5.50 ± 1.50/5.00 ± 1.50	Mesalazine + DanShen injection (4 mL,qd/bid,ivgtt)	Mesalazine (1g, qid,po)	1month	1/2/3
Yang and Wang. (2017)	60 (30/30)	16:14/17:13	40.2 ± 8.8/39.3 ± 6.7	4.7 ± 0.9/4.5 ± 1.2	Mesalazine + DanShen injection (20 mL,qd, ivgtt)	Mesalazine (1g, tid,po)	4weeks	4
Zhang. (2015)	60 (30/30)	18:12/15:15	46.5 ± 8.5/46.8 ± 7.3	5.2 ± 1.8/5.1 ± 2.0	Mesalazine + Tanshinone capsules (1g, tid,po)	Mesalazine (0.8g, tid,po)	4weeks	4/5/6
Zhang. (2020)	60 (30/30)	18:12/17:13	44.59 ± 3.27/44.51 ± 3.24	2.05 ± 0.68/2.09 ± 0.73	Mesalazine + Tanshinone ⅡA injection (80mg, qd, ivgtt)	Mesalazine (1g, qid,po)	7days	1/2/3/4
Zhao. (2021)	80 (40/40)	24:16/22:18	34.60 ± 4.37/34.10 ± 4.25	Unclear	Mesalazine + Tanshinone capsules (1g, tid/qid,po)	Mesalazine (1g, qid,po)	60	5
Zhu. (2018)	54 (27/27)	15:12/16:11	38.53 ± 10.37/39.84 ± 9.68	2.51 ± 1.74/2.75 ± 1.28	Mesalazine + DanShen injection (10 mL,qd, ivgtt)	Mesalazine (1g, qid,po)	4weeks	1/2/3
Zhu. (2019)	98 (49/49)	28:21/29:20	37.4 ± 5.7/37.6 ± 5.8	4.2 ± 0.9/4.2 ± 0.8	Mesalazine + DanShen injection (20 mL,qd, ivgtt)	Mesalazine (1g, tid,po)	4weeks	1/3/5

Note: (1, TNF-α; 2, IL-6; 3, IL-8; 4, CRP; 5, PLT; 6, MPV).

**TABLE 2:** Characteristics of the included animal experimental studies.

Author	Sample size (E/C)	Species	Weight(g)	UC models	Experimental (E)	Control (C)	Treatment duration	Outcomes measures
Chen. (2005)	10/10	rat	210 ± 10	TNBS	Salvianic acid A (2 mL)	Saline	4weeks	1
Fan. (2022)	10/10	mice	20 ± 2	DSS	Cryptotanshinone (60 mg/kg)	NO	28days	1
Guo. (2018)	12/10	mice	19 ± 1	DSS	Dihydrotanshinone I (25 mg/kg)	NO	10days	1/2
Liu. (2020b)	15/15	rat	210 ± 10	TNBS	Tan ⅡA (200 mg/kg)	Saline	7days	2
Peng, 2020	10/10	mice	23 ± 1	DSS	total phenolic acids and tanshinones (100 mg/kg)	Water	7days	1/2
Su. (2021)	7/7	mice	NO	DSS	DanShen (50 mg/kg)	Saline	7days	1/2
Wu. (2021)	10/10	mice	20 ± 2	DSS	Tan ⅡA (60 mg/kg)	0.5% CMC Na solution	7days	1/2
Zhou. (2017)	10/10	mice	22.5 ± 2.5	DSS	Tan ⅡA (200 mg/kg)	NO	7days	1/2
Zhou. (2021)	10/10	mice	20 ± 2	DSS	DanShen (455 mg/kg)	NO	7days	1/2
Zhu. (2021)	10/10	mice	17.5 ± 1.5	DSS	Tanshinol (30 mg/kg)	Saline	14days	1/2
Zhu. (2022)	6/6	mice	21 ± 1	DSS	Tan ⅡA (60 mg/kg)	0.5% CMC Na solution	7days	1/2

Note: (1, TNF-α; 2, IL-6; 3, IL-8; 4, CRP; 5, PLT; 6, MPV).

**TABLE 3:** Reported specific outcomes from clinical studies and animal studies.

Outcomes	Number	Studies
TNF-α	25	Chen, 2005; Chen, 2018; Deng, 2016; Du, 2019; Fan, 2022; Guo, 2018; Li, 2016; Li, 2019; Liang, 2017; Li and Tian, 2019; Peng, 2020; Su, 2021; Wang, 2018b; Wang, 2020a; Wang, 2022; Wu, 2021b; Xiong and Wang, 2016; Xu, 2020; Zhang, 2020; Zhou, 2017; Zhou, 2021; Zhu, 2018; Zhu, 2019; Zhu, 2021; Zhu,2022
effective rate	22	Chen, 2018; Deng, 2016; Du, 2019; Li, 2016; Li, 2019; Liang, 2017; Li and Tian, 2019; Liu, 2011; Liu, 2018; Sun, 2019; Wang, 2018a; Wang, 2020a; Wang, 2020b; Wang, 2022; Xin, 2015; Xu, 2015; Xu, 2020; Yang and Wang, 2017; Zhang, 2020; Zhao, 2021; Zhu, 2018; Zhu, 2019
IL-6	20	Chen, 2018; Deng, 2016; Guo, 2018; Li, 2019; Liang, 2017; Li and Tian, 2019; Liu, 2020; Peng, 2020; Su, 2021; Wang, 2018b; Wang, 2022; Wu, 2021; Xiong and Wang, 2016; Xu, 2020; Zhang, 2020; Zhou, 2017; Zhou, 2021; Zhu, 2018; Zhu, 2021; Zhu,2022
PLT	17	Deng, 2016; Du, 2019; He, 2018a; He, 2018b; Li, 2016; Liang, 2017; Liu, 2011; Liu, 2018; Wang, 2018b; Wang, 2020a; Wang, 2020b; Wang, 2022; Xiong and Wang, 2016; Xu, 2015; Zhang, 2015; Zhao, 2021; Zhu, 2019
IL-8	12	Deng, 2016; Du, 2019; Li, 2016; Liang, 2017; Li et al., 2019; Wang, 2018b; Wang, 2020a; Wang, 2022; Xu, 2020; Zhang, 2020; Zhu, 2018; Zhu, 2019
MPV	11	Deng, 2016; Du, 2019; He, 2018a; He, 2018b; Li, 2016; Liang, 2017; Liu, 2018; Wang, 2020b; Wang, 2022; Xiong and Wang, 2016; Zhang, 2015
CRP	9	He, 2018a; He, 2018b; Sun, 2019; Xin, 2015; Xiong and Wang, 2016; Xu, 2015; Yang and Wang, 2017; Zhang, 2015; Zhang, 2020

A total of 218 animals were included in animal experimental studies studies. All animals in the experimental group was 110 and that in the control group was 108. Animal species including rat and mice were included in meta-analysis. The weight of rats ranged from 200 to 220 g in all studies and that of mice ranged from 15 to 25 g, one study did not report the weight of animals. There were two types of animal models in these studies, namely, TNBS-induced UC in rats and DSS-mediated UC in mice.

### 3.3 Characteristics of bias analysis of included studies

**FIGURE 2:**
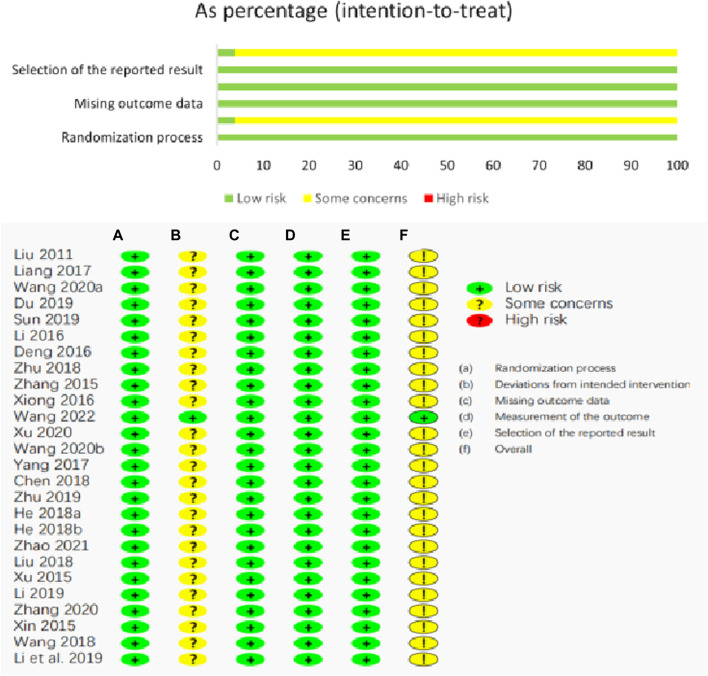
Risk of bias assessment of included clinical studies for this review **(A)** Randomization process; **(B)** Deviations from intended intervention; **(C)** Missing outcome data; **(D)** Measurement of the outcome; **(E)** Selection of the reported result; **(F)** Overall.

All studies mentioned random grouping. The quality of the included literature was evaluated according to the Risk of Bias Table recommended by the Cochrane Manual (ROB-2 tool) and SYRCLE’s RoB tool. The specific results are shown in Figure 2 and Table 4.

**TABLE 4:** Risk of bias of included animal experimental studies.

Author	(1)	(2)	(3)	(4)	(5)	(6)	(7)	(8)	(9)	(10)
Chen, 2005	Y	U	U	U	U	U	N	Y	Y	U
Fan, 2022	Y	U	U	Y	U	U	N	Y	Y	U
Guo, 2018	Y	U	U	U	U	U	N	Y	Y	U
Liu, 2020	Y	U	U	U	U	U	N	Y	Y	U
Peng, 2020	U	U	U	Y	U	U	N	Y	Y	U
Su, 2021	U	U	U	Y	U	U	N	Y	Y	U
Wu, 2021	Y	U	U	U	U	U	N	Y	Y	U
Zhou, 2017	Y	U	U	U	U	U	N	Y	Y	U
Zhou, 2021	Y	U	Y	Y	U	U	N	Y	Y	U
Zhu, 2021	Y	U	U	Y	U	U	N	Y	Y	U
Zhu, 2022	Y	U	U	Y	U	U	N	Y	Y	U

Note: 1) sequence generation; 2) baseline characteristics; 3) allocation concealment; 4) random housing; 5) blinding (performance bias); 6) random outcome assessment; 7) blinding (detection bias); 8) incomplete outcome data; 9) selective outcome reporting; 10) other sources of bias; Y, yes; N, no; U, unclear.

### 3.4 Methodological quality of included studies

The 37 included studies were evaluated using the Cochrane Collaboration tool, with two independent reviewers assessing the quality of each study and a third reviewer resolving disagreements.

### 3.5 Primary outcomes

#### 3.5.1 Effective rate

**FIGURE 3:**
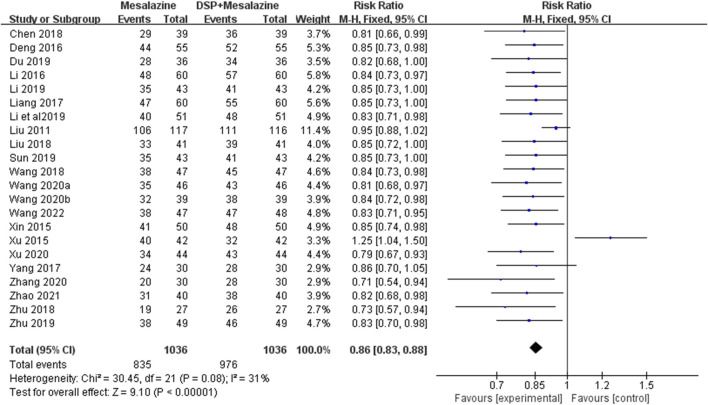
A meta-analysis of clinical efficacy rate in clinical studies of DSP + mesalazine in the treatment of UC.

Twenty-two clinical studies (Liu (2011); Xin et al., 2015; Xu, 2015; Deng et al., 2016; Liang et al., 2017; Yang and Wang, 2017; He, 2018a; He, 2018b; Wang et al., 2018b; Chen et al., 2018; Liu et al., 2018; Zhu, 2018; Du, 2019; Li, 2019; Sun, 2019; Zhu, 2019; Wang, 2020a; Wang, 2020b; Xu, 2020; Zhang, 2020; Zhao, 2021; Wang, 2022) reported effective rate, heterogeneity test results were *p* = 0.08 and I^2^ = 31%, indicating that there was no obvious heterogeneity among the results. Using a fixed-effect model, the analysis showed statistically significant levels of efficiency (N = 2072, RR 0.86, 95% CI:0.83–0.88, *p* < 0.00001), indicating that the combination of DSP and mesalazine was clinically more effective than mesalazine alone Figure 3.

#### 3.5.2 PLT

**FIGURE 4:**
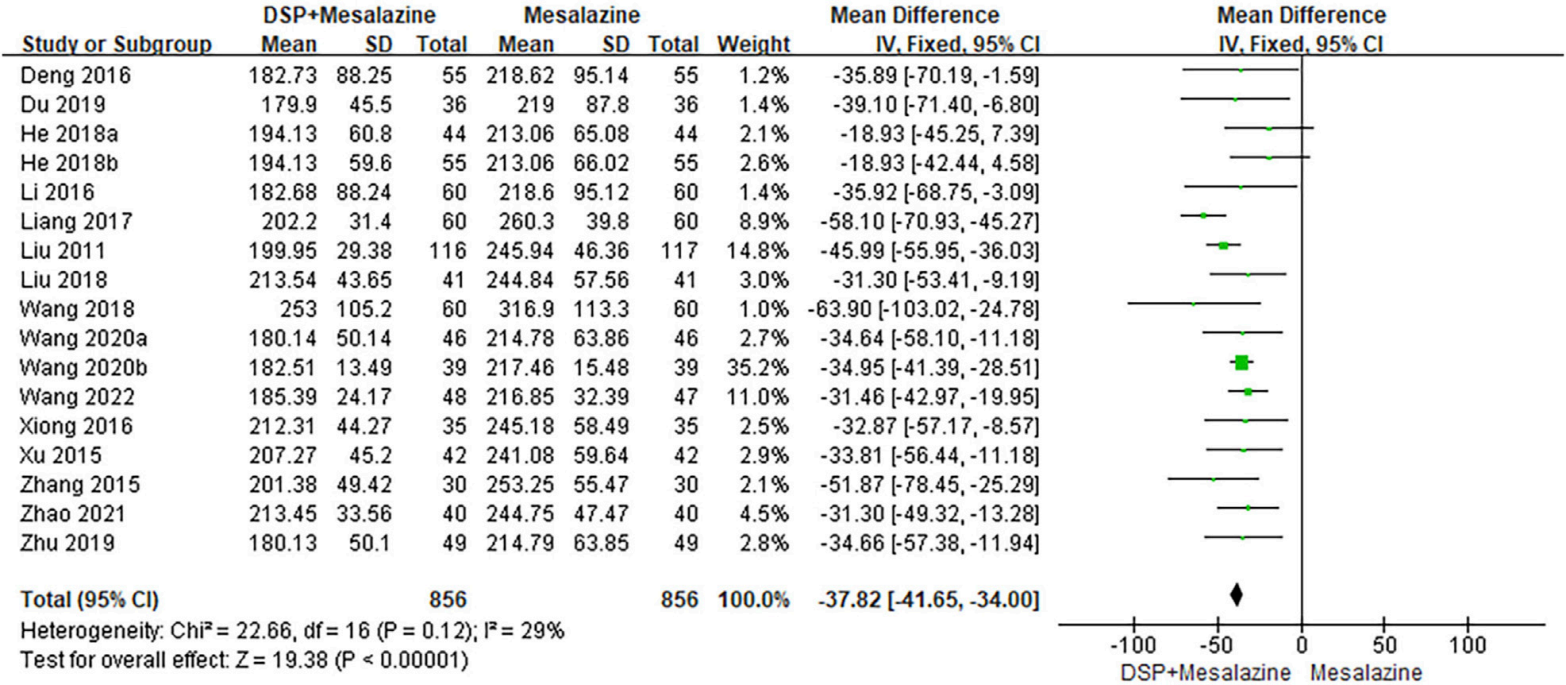
A meta-analysis of PLT in clinical studies of DSP + mesalazine in the treatment of UC.

Seventeen clinical studies (Liu (2011); Xu, 2015; Zhang et al., 2015; Deng et al., 2016; Li, 2016; Xiong and Wang, 2016; Liang et al., 2017; He, 2018a; He, 2018b; Wang et al., 2018b; Liu et al., 2018; Du, 2019; Zhu, 2019; Wang, 2020a; Wang, 2020b; Zhao, 2021; Wang, 2022) reported PLT. No significant heterogeneity (*p* = 0.12, I^2^ = 29%) was seen between the same tests and the analysis showed that PLT levels (N = 1712, SMD -37.82%, 95% CI: −41.65 to −34.00, *p* < 0.00001) were statistically significant, indicating that PLT decreased better in the experimental group than in the control group after treatment (Figure 4).

#### 3.5.3 MPV

**FIGURE 5:**
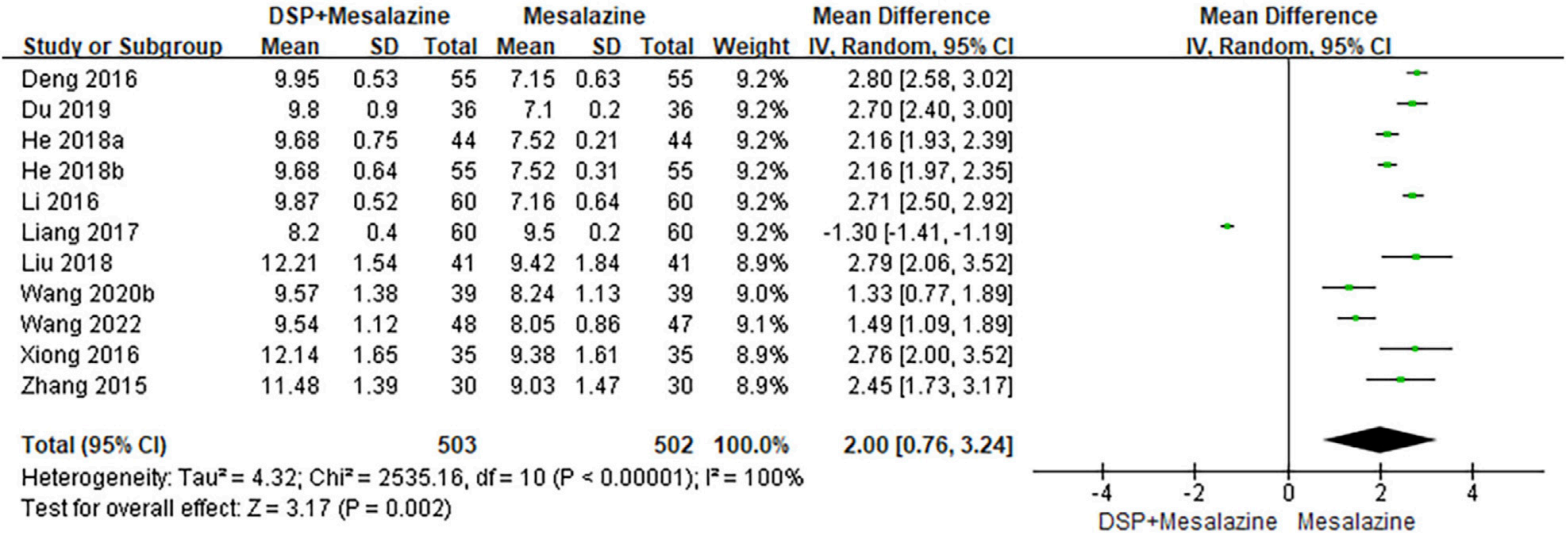
A meta-analysis of MPV in clinical studies of DSP + mesalazine in the treatment of UC.

Eleven clinical studies (Zhang et al., 2015; Deng et al., 2016; Li, 2016; Xiong and Wang, 2016; Liang et al., 2017; He, 2018a; He, 2018b; Liu et al., 2018; Du, 2019; Wang, 2020b; Wang, 2022) reported MPV. As a result of the obvious heterogeneity (*p* < 0.00001, I^2^ = 100%) between the same tests, a random effect model was adopted. The analysis showed a statistically significant increase MPV levels (N = 1005, SMD 2.00%, 95% CI:0.76 to 3.24, *p* = 0.002), suggesting that the combination of DSP and mesalazine was superior to Mesalazine alone after treatment (Figure 5). Subgroup analysis showed that the effects of different time of administration (*p* = 0.08, I^2^ = 60.7%) and different DSP components (*p* = 0.53, I^2^ = 0%) on CRP levels were not significant, while the effects of different doses (*p* = 0.03, I^2^ = 71.3%) of administration were opposite (Table 5).

**TABLE 5:** Results of subgroup analysis.

Number of effect sizes	WMD(95%CI)	*p*-value	Heterogeneity		
			P Heterogeneity	I^2 (%)^	P Between sub-groups
**MPV**					
Overall effect 11					
Treatment duration					
<1 month 2	2.16 [2.01, 2.31]	<0.00001	1.00	0	0.08
= 1 month 7	1.74 [−0.05, 3.52]	0.06	<0.00001	100	
>1month 2	2.78 [2.25, 3.30]	<0.00001	0.96	0	
chemical components					
DanShen 6	1.62 [−0.32, 3.56]	0.10	<0.00001	100	0.53
Tanshinone 5	2.25 [2.06, 2.44]	<0.00001	0.26	24	
dosages					
injection≥ 20 5	1.41 [−0.77, 3.58]	0.21	<0.00001	100	0.03
injection <20 1	2.70 [2.40, 3.00]				
peros = 1 5	2.25 [2.06, 2.44]	<0.00001	0.26	24	
**CRP**					
Overall effect 9					
treatment duration					
<1 month 4	−5.17 [−5.60,−4.74]	<0.00001	1.00	0	0.21
= 1 month 3	−3.84 [−5.67, −2.01]	<0.00001	<0.00001	93	
>1 month 2	−4.25 [−5.75, −2.75]	<0.00001	0.10	63	
chemical components					
Dan Shen 2	−4.57 [−6.02, −3.12]	<0.00001	0.05	75	0.91
Tanshinone 7	−4.47 [−5.50, −3.67]	<0.00001	<0.00001	86	
dosages					
injection≥ 20 3	−4.20 [−5.33, −3.07]	<0.00001	0.39	0	0.19
injection <20 1	−5.20 [−5.84, −4.56]				
peros = 1 5	−4.30 [−5.45, −3.14]	<0.00001	<0.00001	90	
**TNF-α in clinical studies**					
Overall effect 15					
treatment duration					
<1 month 4	−26.24 [−41.30, −11.19]	0.0006	<0.00001	98	<0.00001
= 1 month 10	−50.50 [−59.40, −41.60]	<0.00001	<0.00001	94	
>1 month 1	−8.97 [−10.78, −7.16]				
chemical components					
Dan Shen 12	−46.63 [−58.90, −34.37]	<0.00001	<0.00001	98	0.001
Tanshinone 3	−19.32 [-30.82,-7.82]	0.001	<0.00001	99	
dosages					
injection≥ 20 11	−38.35 [−50.19, −26.50]	<0.00001	<0.00001	98	<0.00001
injection< 20 3	−63.47 [-94.60,-32.35]	<0.0001	<0.00001	98	
peros = 1 1	−8.97 [−10.78, −7.16]				
**TNF-α in animal studies**					
Overall effect 10					
species					
mice 9	−112.04 [−141.36,-82.72]	<0.00001	<0.00001	98	<0.00001
rats 1	−2600 [-2861.57, −2338.43]				
treatment duration					
>7 days 4	−620.67 [-850.48,-390.87]	<0.00001	<0.00001	99	<0.0001
7 days 6	−104.29 [−136.10, −72.47]	<0.00001	<0.00001	98	
dosages					
<100 mg/kg 6	−138.58 [−192.92, −84.23]	<0.00001	<0.00001	98	<0.00001
≥100 mg/kg 3	−84.93 [−125.48, −44.39]	<0.00001	<0.00001	99	
2 mL 1	−2600 [−2861.57, −2338.43]				
**IL-6 in clinical studies**					
Overall effect 11					
treatment duration					
<1 month 4	−19.10 [−29.41, −8.79]	0.0003	<0.00001	99	<0.00001
= 1 month 6	−25.00 [−36.32, −13.67]	<0.0001	<0.00001	98	
>1 month 1	−39.94 [−51.98, −27.90]	<0.00001			
chemical components					
Dan Shen 8	−22.27 [−29.22, −15.33]	<0.00001	<0.00001	97	0.62
Tanshinone 3	−28.72 [−53.57, −3.86]	0.02	<0.00001	99	
dosages					
injection≥ 20 8	−21.05 [−29.32, −12.78]	<0.00001	<0.00001	99	<0.00001
injection< 20 2	−29.24 [−34.49, −23.99]	<0.00001	1.00	0	
peros = 1 1	−39.94 [-51.98,-27.90]	<0.00001			
**IL-6 in animal studies**					
Overall effect 9					
species					
mice 8	−60.33 [−64.59, −56.06]	<0.00001	<0.00001	98	0.10
rats 1	−83.70 [−111.01, −56.39]				
dosages					
<100 mg/kg 5	−77.85 [−84.52, −71.17]	<0.00001	0.02	66	<0.00001
≥100 mg/kg 4	−49.63 [−55.07, −44.20]	<0.00001	<0.00001	99	
treatment duration					
>7 days 2	−78.82 [−85.98, −71.66]	<0.00001	<0.00001	99	<0.00001
7 days 7	−51.37 [−56.58, −46.16]	<0.00001	0.40	0	
**IL-8**					
Overall effect 12					
treatment duration					
<1 month 3	−209.72 [−422.60, 3.16]	0.05	<0.00001	99	0.99
= 1 month 9	−210.93 [−247.38, −174.48]	<0.00001	<0.00001	99	
chemical components					
Dan Shen 11	−227.68 [−263.28, −192.08]	<0.00001	<0.00001	99	<0.00001
Tanshinone 1	−35.08 [-45.76,-24.40]	<0.00001			
dosages					
injection≥ 20 9	−179.54 [−211.63, −147.45]	<0.00001	<0.00001	98	0.48
injection <20 3	−282.17 [−562.47, −1.88]	0.05	<0.00001	99	

#### 3.5.4 CRP

**FIGURE 6:**
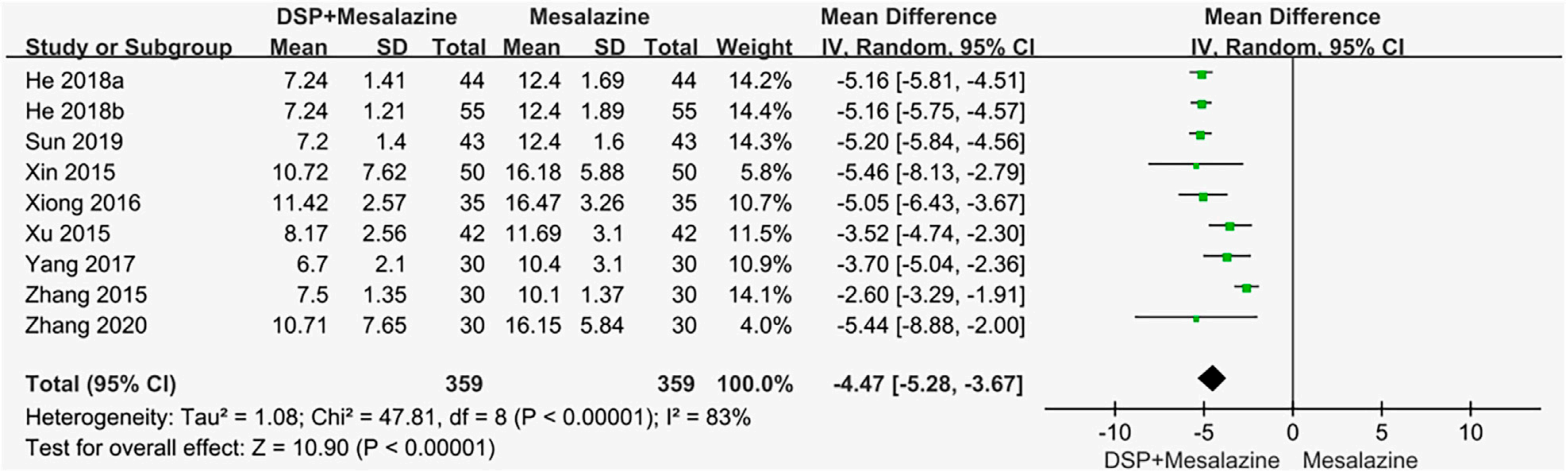
A meta-analysis of CRP in clinical studies of DSP + mesalazine in the treatment of UC.

Nine clinical studies (Xin et al., 2015; Xu, 2015; Zhang et al., 2015; Xiong and Wang, 2016; Yang and Wang, 2017; He, 2018a; He, 2018b; Sun, 2019; Zhang, 2020; Wang, 2022) reported CRP, there was significant heterogeneity among different tests (*p* < 0.00001, I^2^ = 83%). Therefore a random effects model was adopted. Statistically significant reduction in CRP levels (N = 718, SMD −4.47, 95% CI: −5.28 to −3.67, *p* < 0.00001) was observed after treatment with DSP and mesalazine, showing that their combination was superior to mesalazine alone (Figure 6). In none of the subgroups mentioned above, different timing of medication different components of DSP and different administration doses on CRP levels (Table 5).

### 3.6 Secondary outcomes

#### 3.6.1 TNF-α

**FIGURE 7:**
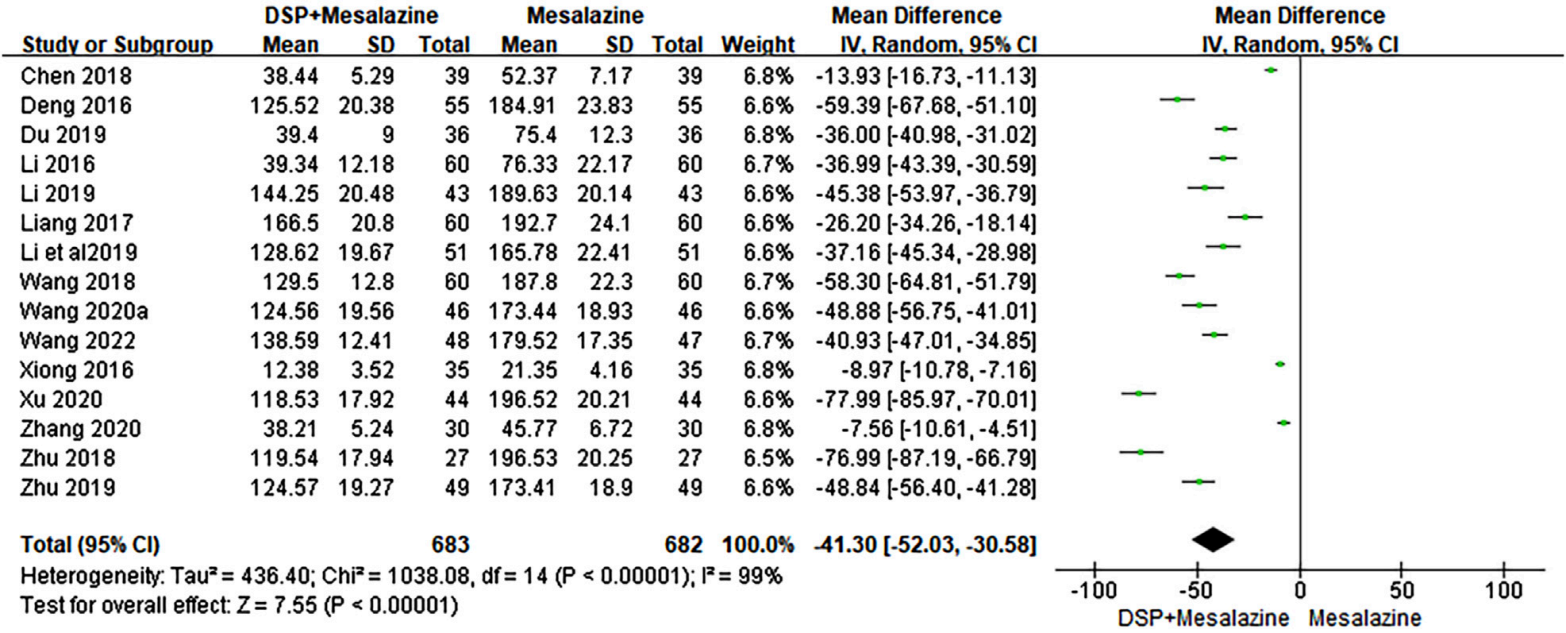
A meta-analysis of TNF-α in clinical studies of DSP + mesalazine in the treatment of UC.

Fifeen clinical studies (Deng et al., 2016; Li, 2016; Xiong and Wang, 2016; Liang et al., 2017; Wang et al., 2018b; Chen et al., 2018; Zhu, 2018; Du, 2019; Li, 2019; Li and Tian, 2019; Zhu, 2019; Wang, 2020a; Xu, 2020; Zhang, 2020; Wang, 2022) reported TNF-α. The random-effects model was chosen because of significant heterogeneity (*p* < 0.00001, I^2^ = 99%) between tests. The difference in TNF-α levels (N = 1365, SMD −41.30, 95%CI: −52.03 to −30.58, *p* < 0.00001) after treatment was statistically significant, implying that in the control group was not as good as that in the experimental group after treatment (Figure 7). Subgroup analysis showed significant effects of different chemical composition (*p* = 0.001, I^2^ = 90.1%), administration time (*p* < 0.0001, I^2^ = 97.6%) and administration dose (*p* < 0.00001, I^2^ = 94.2%) on TNF-α levels (Table 5).

**FIGURE 8:**
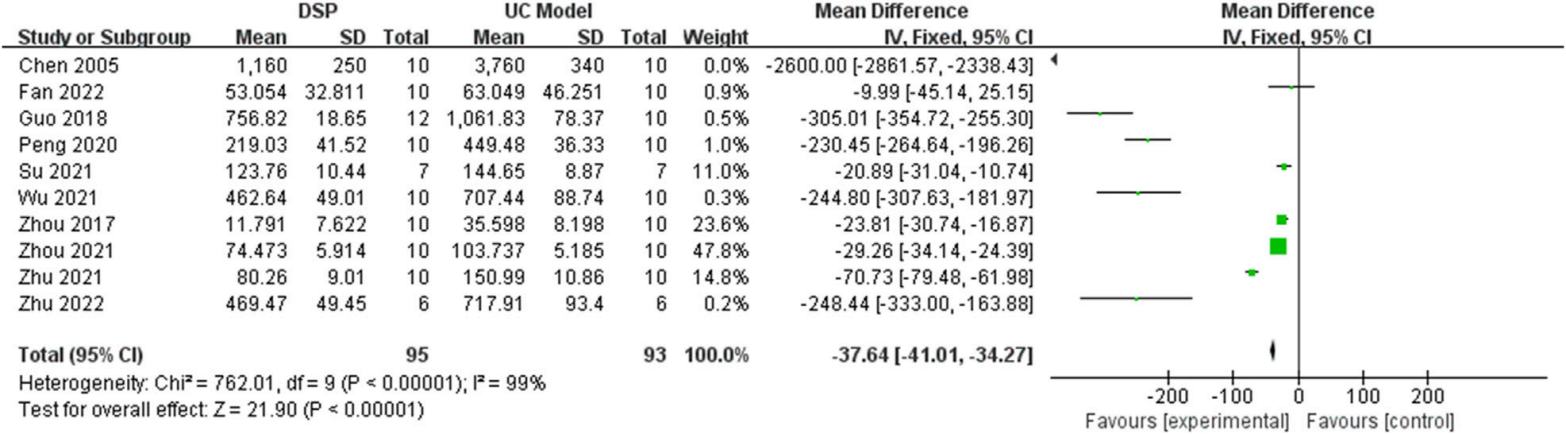
A meta-analysis of TNF-α in animal experimental studies of DSP in the treatment of UC.

Ten animal experimental studies (Chen et al., 2005; Zhou et al., 2017; Guo et al., 2018; Peng et al., 2021b; Wu et al., 2021; Su et al., 2021; Zhu et al., 2021; Fan et al., 2022; Zhou, 2022; Zhu et al., 2022) reported TNF-α. The random-effects model was chosen because of significant heterogeneity (*p* < 0.00001, I^2^ = 99%) between tests. The pooled effect sizes indicated that DSP could significantly decrease levels compared with the control group (SMD −37.64, 95%CI: −41.01 to −34.27, *p* < 0.00001) (Figure 8).Subgroup analysis showed significant effects of different species (*p* < 0.00001, I^2^ = 99.7%), administration time (*p* < 0.0001, I^2^ = 94.7%) and administration dose (*p* < 0.00001, I^2^ = 99.4%) on TNF-α levels (Table 5).

#### 3.6.2 IL-6

**FIGURE 9:**
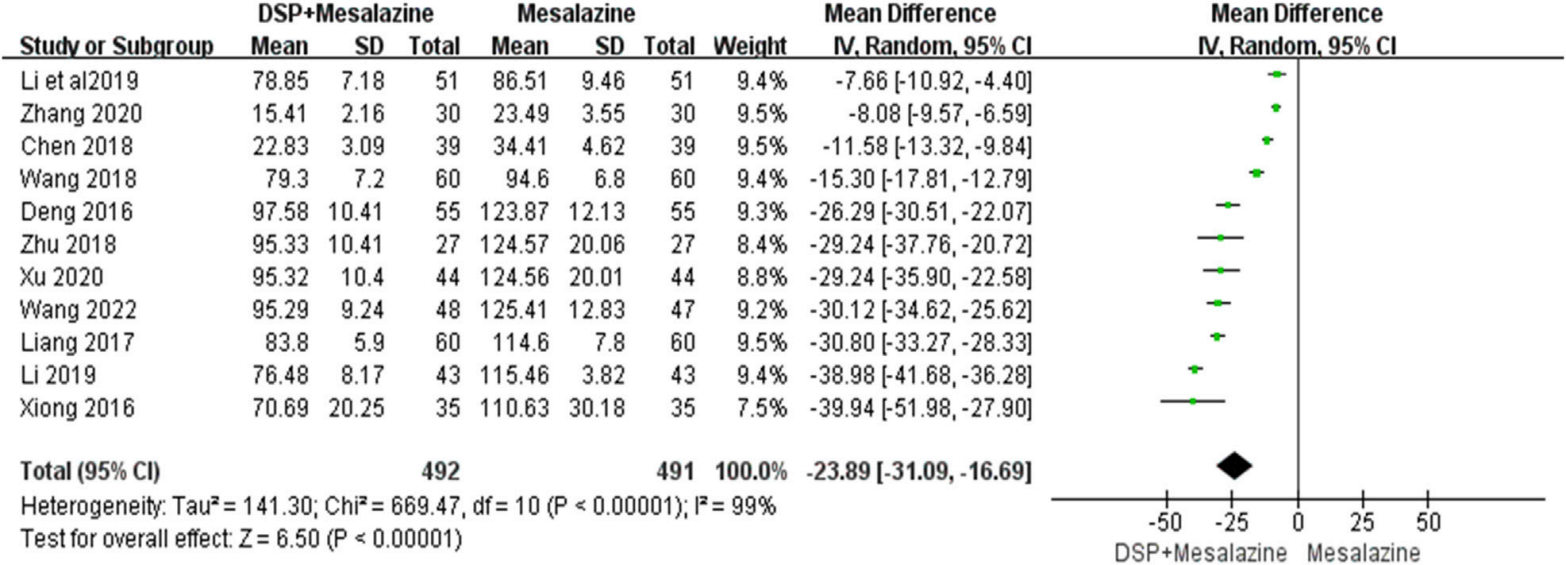
A meta-analysis of IL-6 in clinical studies of DSP + mesalazine in the treatment of UC.

Eleven clinical studies (Deng et al., 2016; Xiong and Wang, 2016; Liang et al., 2017; Wang et al., 2018b; Chen et al., 2018; Zhu, 2018; Li, 2019; Li and Tian, 2019; Xu, 2020; Zhang, 2020; Wang, 2022) reported IL-6. We used a random effects model due to significant heterogeneity between the tests. The analysis showed a statistically significant decrease in IL-6 levels (N = 983, SMD −23.89, 95% CI: −31.09 to −16.69, *p* < 0.00001), suggesting that a better decrease occurred in the experimental group compared with the control group (Figure 9). Subgroup analysis showed that different administration time (*p* < 0.00001, I^2^ = 99%) and administration dose (*p* = 0.04, I^2^ = 70.0%) of DSP had significant effect on IL-6 level, but different components (*p* = 0.62, I^2^ = 0%) had no significant effect (Table 5).

**FIGURE 10:**
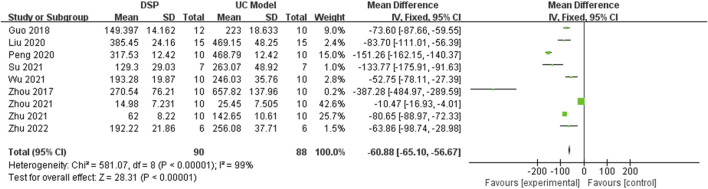
A meta-analysis of IL-6 in animal experimental studies of DSP in the treatment of UC.

Nine animal experimental studies (Zhou et al., 2017; Guo et al., 2018; Liu et al., 2020; Peng et al., 2021b; Wu et al., 2021; Su et al., 2021; Zhu et al., 2021; Zhou, 2022; Zhu et al., 2022) reported IL-6. The random-effects model was chosen because of significant heterogeneity (*p* < 0.00001, I^2^ = 99%) between tests. The pooled effect sizes indicated that DSP could significantly decrease IL-6 levels compared with the control group (SMD −60.88, 95% CI: −65.10 to −56.67, *p* < 0.00001) (Figure 10). Subgroup analysis showed that there were significant differences in administration time (*p* < 0.00001, I^2^ = 97.3%) and dose (*p* < 0.00001, I^2^ = 97.6%) had a significant effect on IL-6 levels. Subgroup analysis showed no significant differences among species (*p* = 0.10, I^2^ = 63.6%) (Table 5).

#### 3.6.3 IL-8

**FIGURE 11:**
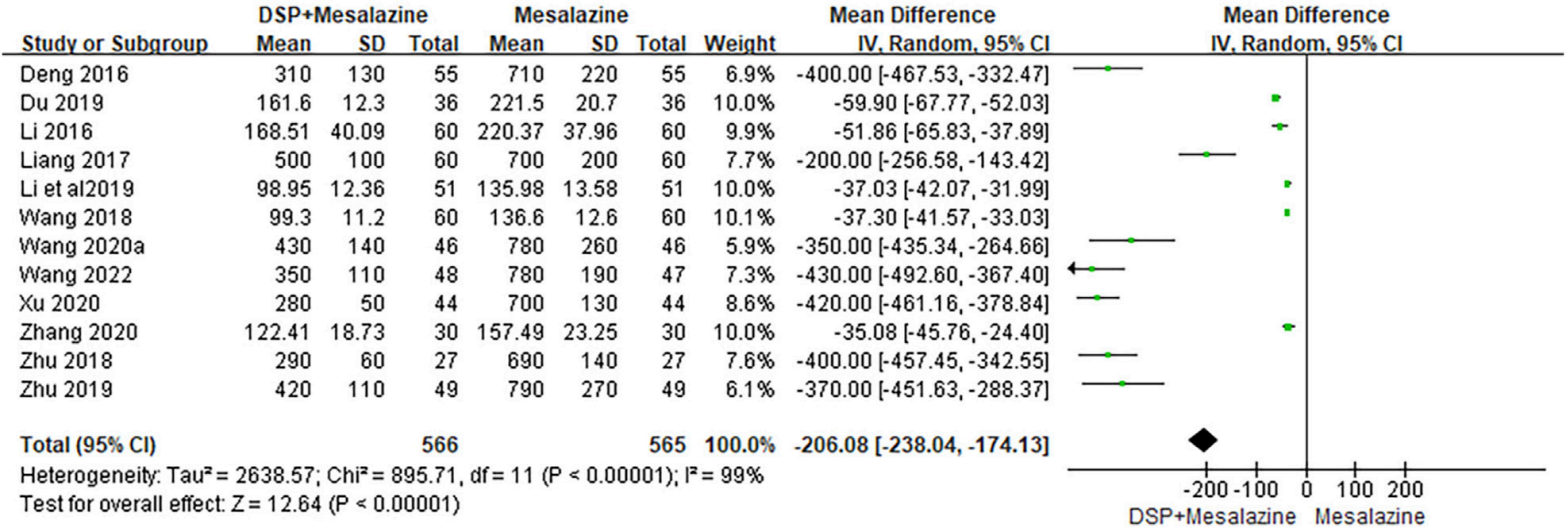
A meta-analysis of IL-8 in clinical studies of DSP + mesalazine in the treatment of UC.

Twelve clinical studies (Deng et al., 2016; Li, 2016; Liang et al., 2017; Wang et al., 2018b; Zhu, 2018; Du, 2019; Li and Tian, 2019; Zhu, 2019; Wang, 2020a; Xu, 2020; Zhang, 2020; Wang, 2022) reported IL-8. A random effects model was used because of significant heterogeneity (*p* < 0.00001, I^2^ = 99%) between the tests. The results of the analysis (N = 1131, SMD −206.08, 95% CI: −238.04 to −174.13, *p* < 0.00001) showed that IL-8 levels was statistically significant, indicating that the experimental group had better IL-8 levels than the control group after treatment (Figure 11). Subgroup analysis showed that different chemical components (*p* < 0.00001, I^2^ = 99%) of DSP significantly affected the level of IL-8, while different administration time (*p* = 0.99, I^2^ = 0%) and species (*p* = 0.48, I^2^ = 0%) had opposite effect (Table5).

### 3.7 Adverse events

**FIGURE 12:**
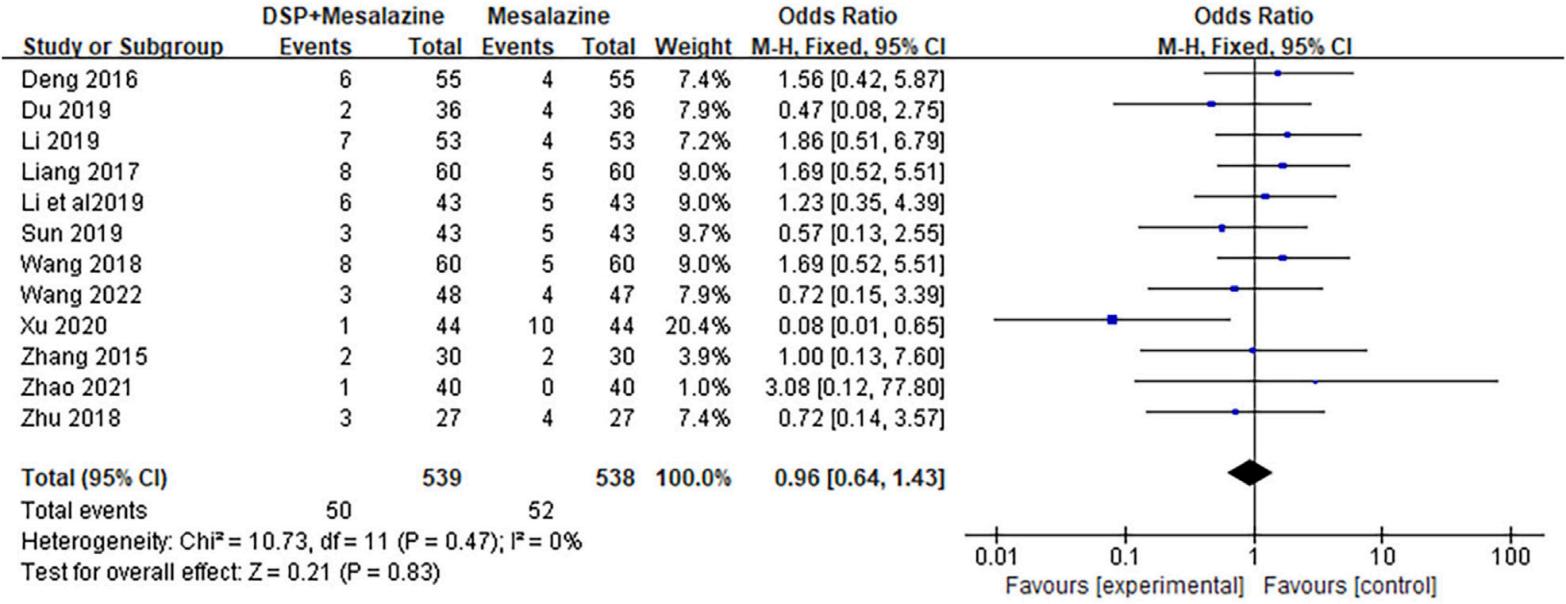
Forest plot of adverse events in clinical studies of DSP + mesalazine in the treatment of UC.

A total of 14 clinical studies (Zhang et al., 2015; Deng et al., 2016; Liang et al., 2017; Wang et al., 2018b; Zhu, 2018; Du, 2019; Li, 2019; Li and Tian, 2019; Sun, 2019; Zhu, 2019; Wang, 2020a; Xu, 2020; Zhang, 2020; Zhao, 2021; Wang, 2022) reported adverse events, including onek (Zhao, 2021) in which no significant adverse events were found in either group during the study. One of the 13 study reports (Zhang, 2020) did not indicate the specific number of adverse events, and the heterogeneity among studies was low (*p* = 0.47, I^2^ = 0%). Random effects model was helpful to analyze adverse reactions. There was no significant difference between the Mesalazine combined DSP group and the Mesalazine single drug group (RR = 0.96, 95% CI = 0.64 to 1.43, *p* = 0.83; Figure 12). Our results showed that nausea, abdominal pain, diarrhea, and pruritus were the most common adverse events. These adverse reactions were mild and could be relieved spontaneously or after symptomatic treatment. No serious adverse events or deaths were reported in the included studies. In general, TCM is relatively safe as an adjunct to western medicine. See the Supplementary Material S2 for details.

### 3.8 Sensitivity analysis

Sensitivity analysis of the above indexes was carried out using the elimination word is repeated twice, with the effects and *p*-value changes observed after exclusion of the included studies in turn. The results showed no significant changes in the effect sizes of the prognostic indicators TNF-α, IL-6, IL-8, effective rate, PLT and MPV after the exclusion of all included studies, confirming the stability and reliability of the meta-analysis.

Based on the sensitivity analysis, the difference in meta-analysis results excluding other studies did not significantly change for the prognostic indicator CRP. However, after excluding Zhang’s (2015) study, the heterogeneity (*p* = 0.17, I^2^ = 32%, N = 658, SMD −4.88, 95% CI: −5.32 to −5.44, *p* < 0.00001) changed significantly.

### 3.9 Publication bias

**FIGURE 13:**
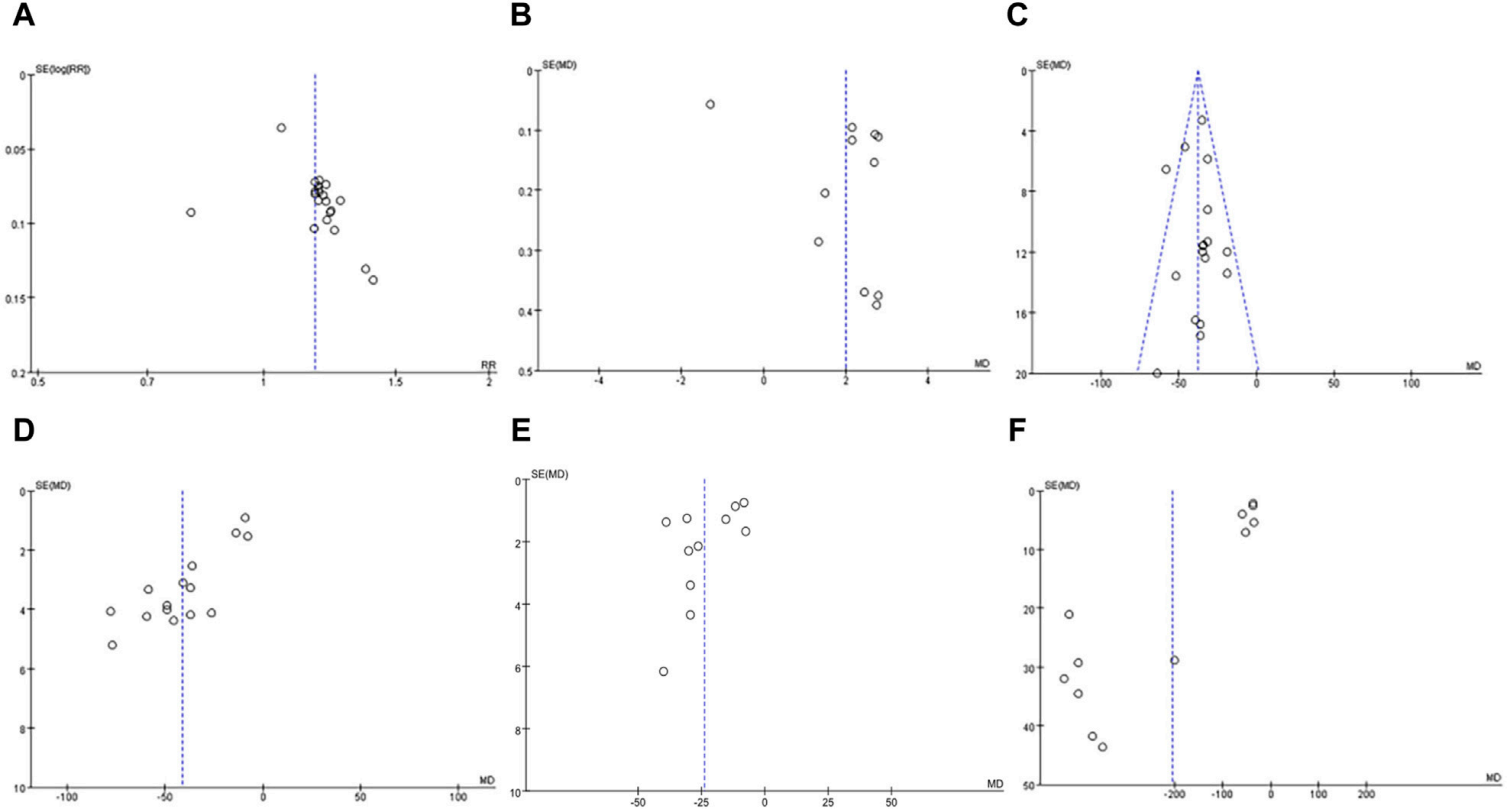
In clinical studies funnel plot of effects of DSP + mesalazine on **(A)** effective rate; **(B)** MPV; **(C)** PLT; **(D)** TNF-α; **(E)** IL-6; **(F)** IL-8.

**FIGURE 14:**
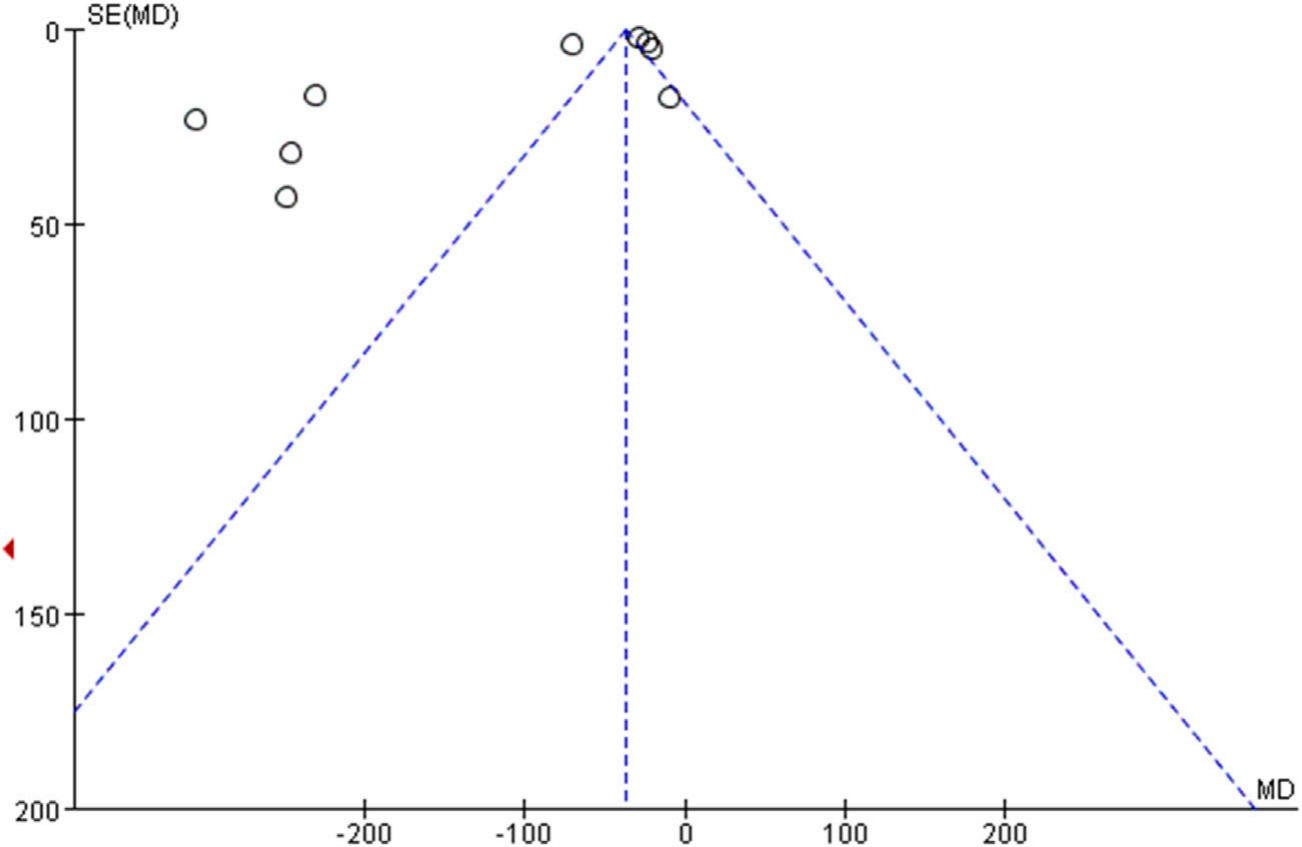
In animal studies funnel plot of effects of DSP on TNF-α.

Funnel plot for evaluating publication bias of 10 or more articles. Therefore, the validity of PLT, effective rate, MPV, TNF-α, IL-6 and IL-8 met the requirements (Figures 13, 14).

(1) effective rate: Through the observation of funnel plot, it was found that the effect of DSP combined with mesalazine on the effective rate may be asymmetric. While the result of Egger’s test was statistically significant. However, Egger’s test (*P* = 0.079) and Begg’s test (*P* = 0.248) found no obvious publication bias.
(2) MPV: Funnel plot observation showed that the effect of DSP combined with mesalazine on MPV levels may be asymmetric. Whereas Egger’s test (*P* = 0.185) and Begg’s test (*P* = 0.029) found obvious publication bias.
(3) PLT: In visual inspection of funnel plots, we found that DSP combined with mesalazine had symmetric effects on PLT levels, whereas Egger’s test (*P* = 0.533) and Begg’s test (*P* = 0.053) did not indicate significant publication bias.
(4) TNF-α: Visual inspection of funnel plots from clinical studies indicated probable symmetrical for the efficacy of DSP combined with mesalazine on TNF-α levels, however, Egger’s test (*P* = 0.001) and Begg’s test (*P* = 0.018) showed significant publication bias.

**FIGURE 15:**
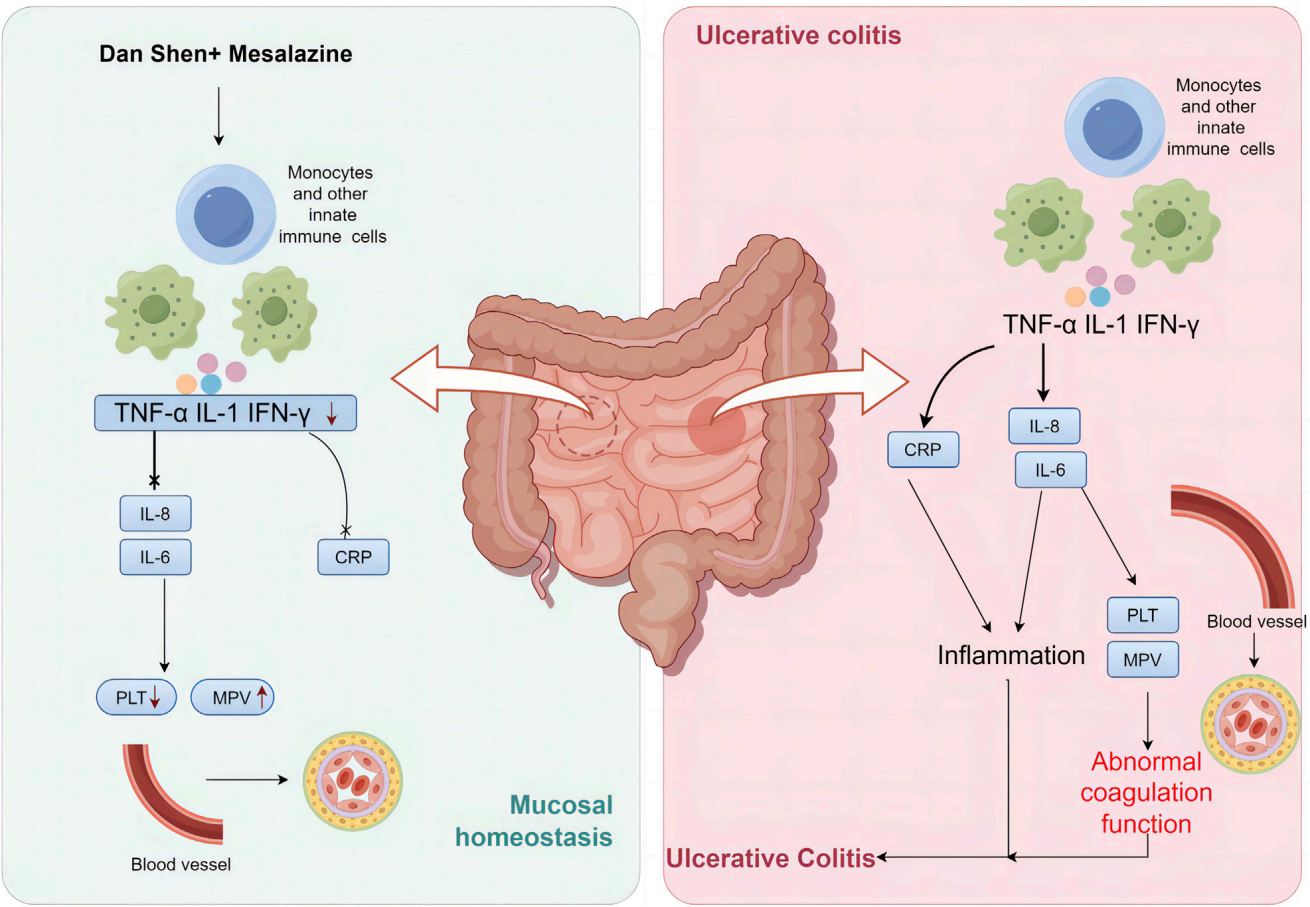
The mechanism of colitis progression and the effect of Danshen against colitis. This figure was drawn in Figdraw.

Visual inspection of funnel plots from animal studies indicated probable symmetrical for the efficacy of DSP on TNF-α levels, however, Egger’s test (*p* = 0.008) and Begg’s test (*p* = 0.049) showed significant publication bias.

(5) IL-6: By observing funnel plot, it was found that the effect of DSP combined with mesalazine on IL-6 levels might be symmetrical, nevertheless, Egger’s test (P = 0.095) and Begg’s test (P = 0.35) demonstrated no significant publication bias.
(6) IL-8: After observation of funnel plot, it was found that the effect of DSP combined with mesalazine on IL-8 level was asymmetric, and this result was supported by Egger’s test (P = 0.000) and Begg’s test (P = 0.011).

## 4 Discussion

A current systematic review and meta-analysis were conducted to evaluate the coagulation function and anti-inflammatory activity of DSP when combined with mesalazine for UC treatment. The results of clinical studies indicate that the combination of mesalazine with DSP significantly improved the clinical efficacy of UC compared to mesalazine alone, a finding supported by animal experiments. Furthermore, it improved coagulation function by reducing PLT levels in patients’ serum and increasing MPV levels, helping control the inflammatory response by reducing CRP, TNF-α, IL-6, and IL-8 levels in patients’ serum. Interestingly, sensitivity analysis, which excluded one study for each stage and response rate, showed that the results for PLT, MPV, TNF-α, IL-6, and IL-8 did not significantly change, whereas Zhang’s study (Zhang et al., 2015) significantly altered the results for CRP. This illustrates that the 95% CI of the results after excluding these studies one by one is less variable, with more overlap, and the results of the meta-analysis are relatively stable, except for CRP.

In recent years, the incidence of UC has stabilized in developed countries such as Europe and the United States, but it is rapidly increasing in several emerging countries in Asia, South America, and the Middle East, posing significant risks to human life (Ha, 2020). Modern medicine remains uncertain about the pathogenesis of UC. Most scholars believe that the pathogenesis of UC is complex and diverse, with research being conducted in the directions of genetics, environment, intestinal microecology, and immune response. As research has progressed, a correlation has been found between UC and coagulation. During the active stage of UC, the body’s blood viscosity increases, coagulation function becomes imbalanced, and the body enters a hypercoagulable state, leading to microthrombosis and impaired microcirculation. This, in turn, results in ischemic necrosis and ulcer formation in the intestinal mucosa, further aggravating the degree of intestinal lesions. Studies have shown that the release of inflammatory factors after platelet activation can promote inflammatory cell infiltration, and inflammatory substances can activate the fibrinolytic-coagulant cascade reaction, resulting in coagulation dysfunction, thus creating a vicious cycle (Polińska et al., 2011; Purnak and Yuksel, 2015; Xu et al., 2017). Danshen is a traditional Chinese medicine known for its ability to promote blood circulation and resolve blood stasis. Pharmacological studies (Dong et al., 2016; Shan et al., 2021) have revealed that Danshen not only improves blood rheology and coagulation but also exhibits anti-inflammatory effects.

Researchers (Tekelioglu et al., 2014; Matowicka, 2016; Gawrońska et al., 2017) have discovered that the number, shape and function of PLT in the serum of UC patients are altered, and that these changes are mainly due to the highly activated state of PLT in the patients’ blood circulation. Meanwhile, most scholars (Lu et al., 2014; Zheng et al., 2018; Zhang et al., 2020) have found a positive correlation between PLT and clinical stage and disease severity of UC, which was also supported by this study. Yüksel’s study (Yüksel et al., 2009) found that MPV was negatively correlated with disease activity in UC, which was consistent with the results of this study. It also examined for the first time the value of MPV in discriminating between active and remitting UC, and concluded that the overall accuracy of MPV in predicting active UC was higher than that of CRP and ESR, implying that MPV is the best indicator for evaluating UC activity. Modern research has shown that the duration of treatment has an effect on the outcome, so it is important to choose the appropriate duration for patient. In this meta-analysis, DSP were available for three different durations (<1 month, 1 month, >1 month), with no significant differences between different treatment courses (*p* = 0.08). Chemical components of DanShen have been widely used in the treatment of UC, such as tanshinone, but there are no relevant studies on the differences in efficacy of different chemical components for treatment. In this meta-analysis, there was no significant difference in the relationship between DanShen and tanshinone at the MPV levels (*p* = 0.53). However, three different administration doses (injection ≥20 mL, injection <20 mL, oral medication 1 g), and the difference was statistically significant (*p* = 0.03).

In our meta-analysis, we discovered that the treatment group was able to significantly reduce PLT and elevate MPV levels, further confirming that DSP combined with mesalazine improved UC by altering intestinal coagulation function. Based on the above findings, we found a crucial problem in the numerous clinical trials, most if not all of which focused on the efficacy of DSP without considering other factors, such as duration of treatment, chemical composition, administered dose, etc. Hence, attention should be paid to the following two aspects in the future clinical trials: Firstly, the conclusion that duration has an effect on the efficacy of DSP needs to be substantiated by more prospective evidence. Secondly, it is necessary to compare the effects of different chemical components in DSP to determine which chemical component is more effective. Thirdly, different administered doses may yield varying levels of bioactive compounds, potentially impacting efficacy.

CRP is an acute phase protein synthesized by hepatocytes in response to inflammatory stimuli such as microbial invasion or tissue damage, which can be used as a marker to evaluate the activity of inflammatory bowel diseases (Sands, 2915). Experimental study (Wang et al., 2022) found that CRP levels were positively correlated with the severity of active UC, which is consistent with the findings of the present study. In our meta-analysis, the difference in CRP levels before and after treatment was statistically significant (*p* < 0.00001), while no significant difference was found in the relationship between course of treatment (*p* = 0.21), chemical composition (*p* = 0.91) and administered dose (*p* = 0.19) in CRP levels in the subgroup analysis. DSP combined with mesalazine significantly reduced CRP levels, implying that the combination suppressed inflammation and improved UC by inhibiting the expression of CRP.

TNF-α is one of the most important pro-inflammatory cytokines and participates in vasodilation, oedema formation and leukocyte adhesion to epithelial cells through the expression adhesion molecules. It regulates blood clotting, contributes to inflammation at sites of oxidative stress and indirectly leads to fever (Zelová and Hošek, 2013; Zhang et al., 2024) Overexpression of IL-6 causes disturbances in the body’s internal environment, affecting electrolyte secretion in the intestinal epithelium and increasing its permeability, which in turn allows neutrophils to escape and infiltrate into inflammatory site (Li et al., 2010). IL-8 is a pro-inflammatory factor produced by monocytes and macrophages, capable of recruiting monocytes and granulocytes to participate in the progression of local inflammation in the intestine (Chapuy et al., 2020). Experimental studies (Kanda et al., 2020; Li et al., 2021; Ge et al., 2022) have demonstrated an increase in colonic tissue TNF-α, IL-6, and IL-8 during the active phase of UC, a fact that was positively correlated with the disease activity of UC, as confirmed by this study.

In this meta-analysis, DSP in combination with mesalazine was found to suppress inflammation and improve UC symptoms by inhibiting TNF-α, IL-6 and IL-8 expression in the treatment group. However, the comparative efficacy of different chemical components on inflammatory factors in UC patients is still unclear. According to our study, DanShen was more effective in improving IL-8 and TNF-α levels compared to tanshinone. On the other hand, there was no apparent difference in IL-6 levels (*p* = 0.62) before and after treatment with various components. These contradictory results may be due to the small sample size in the subgroup analysis. For example, only one study was examined in the IL-8 tanshinone group. In order to further clarify the effects of the chemical composition of DSP on the anti-inflammatory capacity of UC treatment, a larger sample size is necessary. Similarly, there was no measurable difference in IL-8 levels (*p* = 0.99) before and after treatment with different courses of treatment. Treatment duration of 1 month produced the greatest improvement in IL-6 and TNF-α levels compared to both >1 month and <1 month. Again, the reason is that more samples are required to establish the effect of DSP treatment duration on the ability to alleviate inflammation in UC.

Meta-analysis of animal experiments also confirmed this view. It was found that there were significant differences in the levels of TNF-α between different species, different drug time and administration dose. The study found that there were statistically significant differences in IL-6 levels between the time and dose of each drug. However, there was no significant difference in IL-6 level among different species (*p* = 0.1). This may be due to the insufficient sample size.

Based on the results of the meta-analysis, the combination of DSP and mesalazine demonstrated superior efficacy in treating UC compared to mesalazine alone. However, our systematic review and meta-analysis encountered several limitations, including small sample sizes in most included studies, lack of description regarding blinding, and poor methodological quality. Additionally, due to the short intervention duration of up to 2 months, there was insufficient time to evaluate the efficacy and complications of DSP combined with mesalazine throughout the disease course. Moreover, follow-up after treatment was not reported in the included studies. Heterogeneity among the studies may have influenced the assessment of treatment efficacy in the meta-analysis. Furthermore, adverse events were not well-reported in some studies of DSP combined with mesalazine for UC, necessitating a conservative assessment of their safety. These factors could potentially lead to biased results.

## 5 Conclution

The meta-analysis in this systematic review demonstrated that DSP and mesalazine were effective in the treatment of UC by reducing PLT, TNF-α, IL-6, IL-8, CRP, and elevating MPV levels, thereby improving intestinal coagulation function and inflammation levels. Nevertheless, there are some limitations that need to be addressed in the future. Larger sample sizes of randomised controlled trials, higher methodological quality and longer intervention times, and clinical trials involving more countries will help validate the efficacy and safety of DSP in combination with mesalazine in the treatment of UC.

## Data Availability

The raw data supporting the conclusion of this article will be made available by the authors, without undue reservation.

## Abbreviations

DSP, DanShen preparation; DSS, dextran sodium sulfate; UC, Ulcerative Colitis; PLT, platelet count; MPV, mean platelet volume; CRP, C-reactive protein; TNBS, 2, 4, 6-trinitrobenzene sulfonic acid; TNF-α, Tumor necrosis factor-α; IL, Interleukin; CT, conventional treatment; 95% CI, 95% confidence interval; RCTs, randomized controlled trials; PRISMA, Preferred Reporting Items for Systematic Reviews and Meta-analyses; Mesh, Medical subject headings; SMD, standardized mean difference; RR, risk ratio.
