# Supplementary material for: A systematic review and meta-analysis of Danshen combined with mesalazine for the treatment of ulcerative colitis
Source: Front Pharmacol. 2024 May 31;15:1334474. doi: 10.3389/fphar.2024.1334474 (PMC11176616; doi:10.3389/fphar.2024.1334474)
Supplement: Supplementary file 1 [file DataSheet1.docx]

# Supplementary File S1. Search strategies for databases.

|  | **Pubmed** |
| --- | --- |
| Number | Search terms |
| #1 | (Ulcerative Colitis"[MeSH Terms] OR ("colitis"[Title/Abstract] OR "ulcerative"[Title/Abstract]) OR "UC"[Title/Abstract] OR "ulcer colonitis"[Title/Abstract] |
| #2 | ("DanShen"[MeSH Terms] OR "Salvia miltiorrhiza"[Title/Abstract] OR "Tan Seng"[Title/Abstract]) OR "Chinese Salvia"[Title/Abstract] OR "tanshinone"[Title/Abstract] OR "Cryptotanshinone"[Title/Abstract] OR "Salvianolic acid"[Title/Abstract] OR "DanShen preparation"[Title/Abstract] OR "Danshen injection"[Title/Abstract] |
| #3 | "randomized controlled trial"[Title/Abstract] OR "randomised controlled trial"[Title/Abstract] OR "randomized"[Title/Abstract] OR"randomised"[Title/Abstract] OR "RCT"[Title/Abstract] |
| #4 | #1 and #2 and #3 |
|  | **Cochrane Library** |
| #1 | (Ulcerative Colitis[Title/Abstract/keywords]) OR (UC[Title/Abstract/keywords]) OR (ulcer colonitis[Title/Abstract/keywords]) |
| #2 | (DanShen[Title/Abstract/keyword]) OR (Dan Shen[Title/Abstract/keyword]) OR (Dan - Shen [Title/Abstract/keyword])OR (Salvia miltiorrhiza [Title/Abstract/keyword])OR (Salvia - miltiorrhiza [Title/Abstract/keyword]) |
| #3 | ( randomized controlled trial[Title/Abstract]) OR (randomised controlled trial[Title/Abstract]) OR (randomized) OR (randomised [Title/Abstract]) OR (RCT[Title/Abstract]) |
| #4 | #1 and #2 and #3 |
|  | **Web of Science** |
| #1 | (TS = Ulcerative Colitis OR UC OR ulcer colonitis ) |
| #2 | (TS = DanShen OR Dan Shen OR Dan - Shen OR Salvia miltiorrhiza OR Salvia - miltiorrhiza ) |
| #3 | (TS = randomized controlled trial OR randomised controlled trial OR randomized OR randomised OR RCT) |
| #4 | #1 and #2 and #3 |
|  | **Embase** |
| #1 | (Ulcerative Colitis[Title/Abstract/keywords]) OR (UC [Title/Abstract/keywords]) OR ( ulcer colonitis [Title/Abstract/keywords]) |
| #2 | (DanShen[Title/Abstract/keyword]) OR Dan Shen [Title/Abstract/keyword]) OR (Dan - Shen[Title/Abstract/keyword]) OR ( Salvia miltiorrhiza[Title/Abstract/keyword]) OR (Salvia - miltiorrhiza[Title/Abstract/keyword]) |
| #3 | ( randomized controlled trial[Title/Abstract]) OR (randomised controlled trial[Title/Abstract]) OR (randomized[Title/Abstract])OR (randomised[Title/Abstract]) OR (RCT[Title/Abstract]) |
| #4 | #1 and #2 and #3 |
| **China National Knowledge Infrastructure (CNKI)** | |
| (主题=丹参 + 丹参制剂 + 丹参注射液 )  AND  (主题=溃疡性结肠炎 + 结肠炎 + 炎症性肠病 )  AND  (摘要=随机对照 + 随机 + 试验 + RCT) | |
| **China Science and Technology Journal Database (VIP)** | |
| (题名或关键词=丹参 + 丹参制剂 + 丹参注射液)  AND  (题名或关键词=溃疡性结肠炎 + 结肠炎 + 炎症性肠病)  AND  (摘要=随机对照 + 随机 + 试验 + RCT) | |
| **Wanfang Database (Wangfang)** | |
| (题名或关键词=丹参 OR 丹参制剂 OR 丹参注射液)  AND  (题名或关键词=溃疡性结肠炎 OR 结肠炎 OR炎症性肠病 )  AND  (摘要=随机对照 OR 随机 OR 试验 OR RCT) | |
| **China Biology Medicine disc (CMB)** | |
| ("丹参"[标题:智能] OR "丹参制剂"[标题:智能] OR "丹参注射液"[标题:智能])  AND  ( "溃疡性结肠炎"[标题:智能] OR "结肠炎"[标题:智能] OR "炎症性肠病痛"[标题:智能] )  AND  ( "随机对照"[摘要:智能] OR "随机"[摘要:智能] OR "试验"[摘要:智能] OR "RCT"[摘要:智能] | |

# Supplementary File S2. Adverse drug reactions or adverse events.

| Studies | Adverse drug reactions or adverse events | |
| --- | --- | --- |
|  | the experimental group | the control group |
| Deng, 2016 | 3 cases of diarrhea, 1 case of skin pruritus, 2 cases of abdominal pain | 2 cases of diarrhea, 1 case of skin pruritus, 1 case of abdominal pain |
| Du, 2019 | 1 case of skin pruritus, 1 case of diarrhea | 2 cases of skin pruritus, 1 case of diarrhea, 1 case of nausea |
| Li, 2019 | 3 cases of nausea, 1 case of abdominal pain, 3 cases of diarrhea | 1 case of nausea, 2 cases of abdominal pain, 1 case of diarrhea |
| Li et al., 2019 | 3 cases of nausea, 3 cases of dizziness | 2 cases of nausea, 3 cases of dizziness |
| Liang, 2017 | 2 cases of nausea, 1 case of abdominal pain, 3 cases of skin pruritus, 1 case of diarrhea, 1 case of flush | 2 cases of nausea, 1 case of abdominal pain, 1 case of skin pruritus, 1 case of dizziness |
| Sun, 2019 | 1 case of nausea, 1 case of fever, 1 case of feel suffocated | 2 cases of nausea, 1 case of fever, 2 cases of rash |
| Wang, 2018b | 2 cases of nausea, 1 case of abdominal pain, 3 cases of skin pruritus, 1 case of diarrhea, 1 case of flush | 2 cases of nausea, 1 case of abdominal pain, 1 case of skin pruritus, 1 case of dizziness |
| Wang, 2022 | 1 case of nausea, 1 case of diarrhea, 1 case of dizziness | 1 case of nausea, 1 case of skin pruritus, 2 cases of dizziness |
| Xu, 2020 | 1 case of nausea | 3 cases of nausea, 3 cases of skin pruritus, 4 cases of diarrhea |
| Zhao, 2021 | 1 case of mild stomach discomfort | / |
| Zhang, 2015 | 2 cases of mild gastrointestinal reactions such as nausea and vomiting | 2 cases of mild gastrointestinal reactions such as nausea and vomiting |
| Zhang, 2020 | dizziness and nausea | dizziness and nausea |
| Zhu, 2018 | 2 cases of skin pruritus, 1 case of diarrhea | 2 cases of skin pruritus, 1 case of diarrhea, 1 case of nausea |
| Zhu, 2019 | / | / |
